# Supplementary material for: New class of symmetric starlike functions subordinate to the generating function of Gregory coefficients
Source: PLoS One. 2025 May 5;20(5):e0317339. doi: 10.1371/journal.pone.0317339 (PMC12052189; doi:10.1371/journal.pone.0317339)
Supplement: Attachment — Submitted filename: Review Report.pdf [file pone.0317339.s001.pdf]

Title: New class of symmetric starlike functions subordinate to the generating functions of Gregory coefficients

Report: The paper " New class of symmetric starlike functions subordinate to the generating functions of Gregory coefficients " by Khan et al. presents an innovative exploration into the realm of geometric function theory through the application of symmetric functions. The authors introduced a class of symmetric starlike functions subordinate to the generating functions of Gregory coefficients. This study uses a specific mathematical technique (subordination) to define a new class of analytic and symmetric starlike functions. The paper demonstrates rigorous mathematical derivations and proofs, ensuring the correctness and reliability of the results. The authors meticulously derived and explored new findings, such as sharp bounds for problem concerning the coefficients of the family of symmetric starlike functions connected to the generating function of Gregory coefficients by utilizing the notion of functions with positive real component. These problems include first five sharp coefficients bounds and Fekete-Szego problem along with the Hankel determinant of order three. Additionally, they explored the optimal bounds (sharp bounds) for two important functions, the logarithmic function and the inverse function within the same class of symmetric starlike functions.

The study not only introduces new classes but also explores sharp functions for all results and figure 1 show the image of unit disk under the functions of Gregory coefficients. The use of sharp functions and graphical studies adds substantial value to this article. The manuscript is well-organized, with clear definitions, theorems, and proofs. The mathematical formulations and derivations presented are precise and well-founded. The introduction comprehensively covers all the necessary concepts related to this work. Abstract and conclusions covers all reseach work and written clearly. Based on a thorough review, the mathematical procedures appear correct. Overall, the paper presents a good analytical idea and just needs a check for typos and grammar.

Here are some suggested changes:

1. On page 1, line 5: remove the extra “can” from the following sentence” A can can be written as:”
2. On page 4, line 17: Replace “precise bound” with “sharp bounds”. We cannot write precise bounds.
3. On page 4: write “ and “ betwee the equations on line 22 and 23.
4. On page 5: Remove the period in Definition 1.
5. On page 6: In the last line of Lemma 2, it should be like this$|x| \leq 1, |y| \leq 1$.
6. On page 6: In the second line of Lemma 4, replace $0 < A < 1$ with $0 < T_2 < 1$
7. On page 7, line 8: Remove “period” and put the “ comma” after (3.9)-(3.12)
8. On page 11, line 25: “By the inequality (??) of Lemma 3, we have”. Equation number is missing.
9. On page 13, line 6: replace "v" with " using".
10. On page 14, line 2: remove the period
11. The conclusion section lacks clarity on future directions. Please revise and expand the conclusions to include specific future research directions

<u>I recommend making these corrections and thoroughly reviewing the entire manuscript for typo error.</u>

Decision: In summary, the paper by Khan et al. makes a significant contribution to the field of symmetric starlike functions and Geometric Functions Theory. The research work, "New class of symmetric starlike functions subordinate to the generating functions of Gregory coefficients," represents a noteworthy achievement. The paper's mathematical rigor and clear presentation make it a valuable resource for researchers and practitioners in the field.

The article can be published after implementing these minor changes.
