# Supplementary material for: New class of symmetric starlike functions subordinate to the generating function of Gregory coefficients
Source: PLoS One. 2025 May 5;20(5):e0317339. doi: 10.1371/journal.pone.0317339 (PMC12052189; doi:10.1371/journal.pone.0317339)
Supplement: Attachment — Submitted filename: Referee_report.pdf [file pone.0317339.s002.pdf]

REFEREE REPORT

Referee Report on The Article entitled "New class of symmetric starlike functions subordinate to the Generating function of Gregory coefficients "-PONE-D-24-33833

In the current paper, the author introduce and investigate the family of symmetric starlike functions which is related to generating function of Gregory coefficients and determined the sharp bounds for problem concerning the coefficients of the family of symmetric starlike functions connected to the generating function of Gregory coefficients . More over Fekete-Szego problem along with the Hankel determinant of order three, the logarithmic function and the inverse function within the same class of symmetric starlike functions which is related to generating function of Gregory coefficients are also determined.

Suggestions or corrections

1. The authors advised to use the variable as z, u, or t not as γ from 91.1) onwards.
2. There is no novelty why author consider this study and as usual routine procedure they obtained the results.
3. check for typos completely for upper case, lower case, and pulley stop , etc.
4. The results are correctly formulated and could be understand easily;
5. The proofs are correct and clearly presented, and there are no any mistakes in the proofs.

   The authors are advised to add few recent works on nephroid domain and Lemiscate domaina, also the references given below
6. A class of -bi-pseudo-starlike functions with respect to symmetric points associated with Telephone numbers. Afr. Mat. 35, 17 (2024). https://doi.org/10.1007/s13370-023-01159-0.
   The authors purposely avoided the following , they must add these articles which are more related to their study.
7. Initial Coefficient Bounds for Bi-Univalent Functions Related to Gregory Coefficients Mathematics 2023, 11(13), 2857; https://doi.org/10.3390/math11132857
8. Korean J. Math.32(2024), No. 2, pp. 285–295https://dx.doi.org/10.11568/kjm.2024.32.2.285FABER POLYNOMIAL COEFFICIENT ESTIMATES FORANALYTIC BI-UNIVALENT FUNCTIONS ASSOCIATED WITH GREGORY COEFFICIENTS

Recommendation: With the above observation, I recommend that the article for publication in "PLOS ONE" after carrying out the corrections.
