# Supplementary material for: New class of symmetric starlike functions subordinate to the generating function of Gregory coefficients
Source: PLoS One. 2025 May 5;20(5):e0317339. doi: 10.1371/journal.pone.0317339 (PMC12052189; doi:10.1371/journal.pone.0317339)
Supplement: Attachment — Submitted filename: Reviewer 2.pdf [file pone.0317339.s003.pdf]

I would like to extend my sincerest gratitude to the editor and reviewers for their meticulous evaluation, insightful suggestions, and constructive comments, which significantly enhanced the quality and clarity of this article.

Reply to the Reviewer #2:

1. On page 1, line 5: remove the extra “can” from the following sentence” A can can be written as:”
2. On page 4, line 17: Replace “precise bound” with “sharp bounds”. We cannot write precise bounds.
3. On page 4: write “ and “ betwee the equations on line 22 and 23.
4. On page 5: Remove the period in Definition 1.
5. On page 6: In the last line of Lemma 2, it should be like this$|x|\leq 1, |y|\leq 1$.
6. On page 6: In the second line of Lemma 4, replace $0<A<1$ with $0<T2<1$
7. On page 7, line 8: Remove “period” and put the “ comma” after (3.9)-(3.12)
8. On page 11, line 25: “By the inequality (??) of Lemma 3, we have”. Equation number is missing.
9. On page 13, line 6: replace "v" with " using".
10. On page 14, line 2: remove the period

Reply: Dear Respected Professor,

We are pleased to report that we have thoroughly addressed points 1-10 in the revised version. Additionally, we have highlighted all revisions and incorporated new references closely related to our study.

11. The conclusion section lacks clarity on future directions. Please revise and expand the conclusions to include specific future research directions.
Reply: Respected Professor: We have incorporated future directions into the revised version. We believe this revision strengthens our manuscript and look forward to potential acceptance.
